# Metabolomics Reveals the Origins of Antimicrobial Plant Resins Collected by Honey Bees

**DOI:** 10.1371/journal.pone.0077512

**Published:** 2013-10-18

**Authors:** Michael B. Wilson, Marla Spivak, Adrian D. Hegeman, Aaron Rendahl, Jerry D. Cohen

**Affiliations:** 1 Department of Horticultural Science, University of Minnesota, St. Paul, Minnesota, United States of America; 2 Microbial and Plant Genomics Institute, University of Minnesota, St. Paul, Minnesota, United States of America; 3 Department of Entomology, University of Minnesota, St. Paul, Minnesota, United States of America; 4 Department of Plant Biology, University of Minnesota, St. Paul, Minnesota, United States of America; 5 School of Statistics, University of Minnesota, Minneapolis, Minnesota, United States of America; United States Department of Agriculture, Agriculture Research Service, United States of America

## Abstract

The deposition of antimicrobial plant resins in honey bee, *Apis mellifera*, nests has important physiological benefits. Resin foraging is difficult to approach experimentally because resin composition is highly variable among and between plant families, the environmental and plant-genotypic effects on resins are unknown, and resin foragers are relatively rare and often forage in unobservable tree canopies. Subsequently, little is known about the botanical origins of resins in many regions or the benefits of specific resins to bees. We used metabolomic methods as a type of environmental forensics to track individual resin forager behavior through comparisons of global resin metabolite patterns. The resin from the corbiculae of a single bee was sufficient to identify that resin's botanical source without prior knowledge of resin composition. Bees from our apiary discriminately foraged for resin from eastern cottonwood (*Populus deltoides*), and balsam poplar (*P. balsamifera*) among many available, even closely related, resinous plants. Cottonwood and balsam poplar resin composition did not show significant seasonal or regional changes in composition. Metabolomic analysis of resin from 6 North American *Populus spp*. and 5 hybrids revealed peaks characteristic to taxonomic nodes within *Populus*, while antimicrobial analysis revealed that resin from different species varied in inhibition of the bee bacterial pathogen, *Paenibacillus larvae*. We conclude that honey bees make discrete choices among many resinous plant species, even among closely related species. Bees also maintained fidelity to a single source during a foraging trip. Furthermore, the differential inhibition of *P. larvae* by *Populus spp*., thought to be preferential for resin collection in temperate regions, suggests that resins from closely related plant species many have different benefits to bees.

## Introduction

Honey bees, *Apis mellifera*, are highly social insects that live in large colonies (e.g., 50,000 individuals). One cost of social living is an increased rate of disease transmission among individuals, and honey bees are highly prone to a diverse set of pathogens and parasites [1]. Managed populations of honey bees in the U.S. are in serious decline, and there has been a 61% decrease of registered colonies from 1947 to 2008 [2]. This decrease is due, in large part, to unsustainable winter losses [2]–[3] caused by the combined effects of diseases, parasites, pesticides, and nutritional deficiencies [4]–[8]. This is particularly alarming because honey bees are estimated to contribute $15–20 billion dollars annually to U.S. agriculture from pollination services alone [9]. Beekeeping practices and regulatory issues indicate that the most sustainable solutions to problems plaguing bees will be derived from promoting their natural defenses through breeding and habitat enhancement. While honey bees have only 1/3 of the genes involved in individual immunity compared to the solitary insects *Drosophila* (fruit fly) and *Anopheles* (mosquito)[10], they do have a suite of cooperative behaviors that contribute to colony health called ‘social immunity’ [11]–[13]. An example of social immunity is hygienic behavior, where honey bees work together to detect and remove diseased brood from the nest, resulting in colony-level resistance to pathogens and parasites [12]–[15]

**Figure 1:**
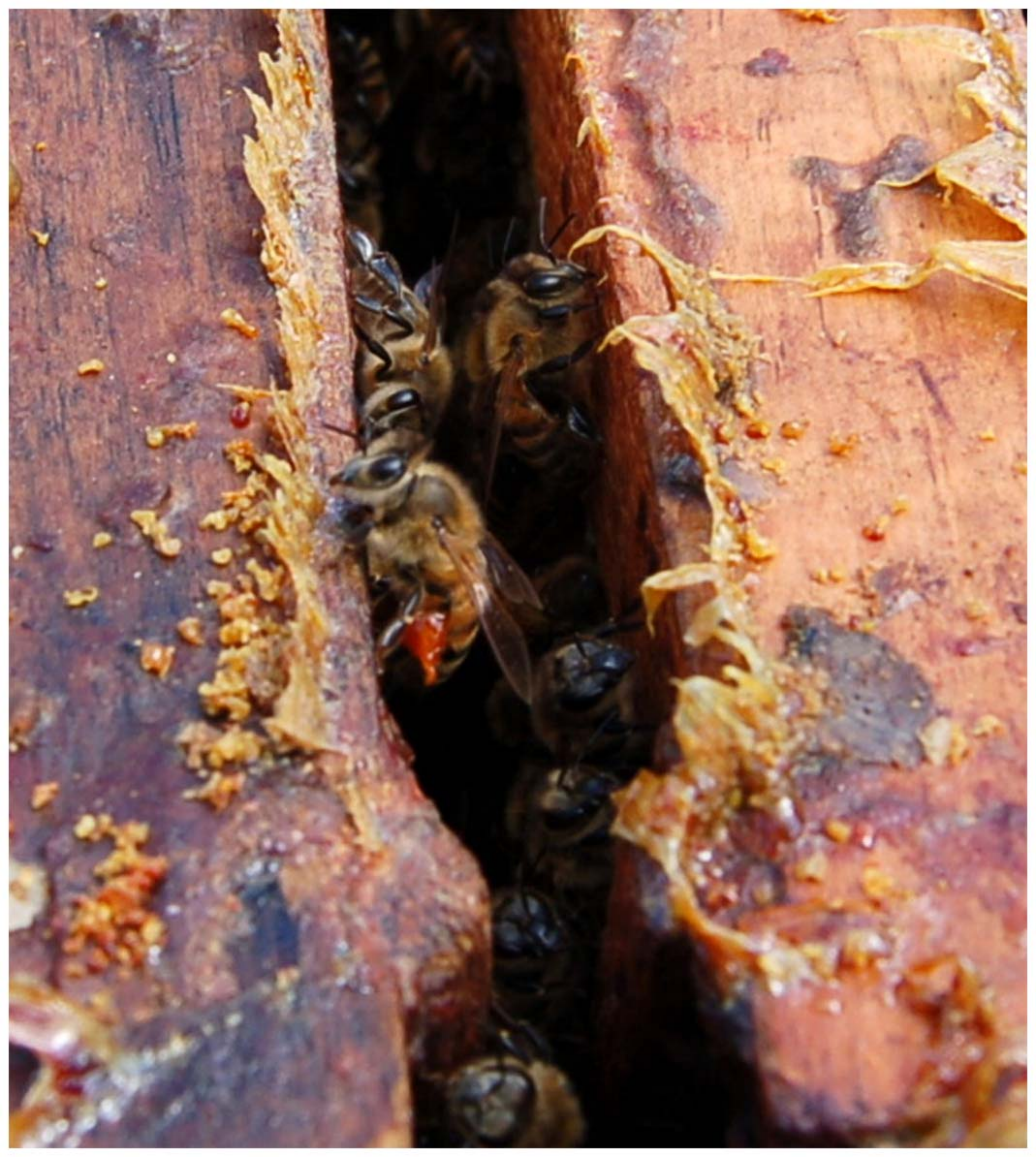
Honey bee resin collection. Top-down view of standard beekeeping equipment displaying a resin forager with red resin attached to her hind legs. Managed honey bees deposit resin mainly at the hive entrance, inner cover, and on top of the movable frames.

In addition to hygienic behavior, bees also deposit antimicrobial plant resins in their nests that have important immunological benefits [16] (Fig. 1). Feral honey bees coat the entire inside surface of their nesting cavity in with resin [17], but managed honey bees deposit comparatively little resin in conventional beekeeping hive boxes. Resins are complex mixtures of phenolic and isoprenoid compounds [18] secreted by plants to provide protection against predators and pathogenic microorganisms [19]–[20]. The chemical composition of resins is complex and variable within and among plant families, traits that makes resin production a good defense against rapidly evolving pests and pathogens [21]. Many organisms, including insects, birds, and humans, collect and use resins to protect against their own pathogens and parasites [22]–[24].

Honey bees collect resins on their hind legs and deposit them in the nest where the resin, often mixed with wax, is called ‘propolis’ by apiculturists. Honey bees are known to collect resin from a wide variety of plants depending on what is available in their environment, though bees in temperate climates are thought to collect mainly from *Populus* (cottonwood, poplar, aspen), but also from *Betula* (birch), *Salix* (willow), *Alnus* (alder), and *Aesculus* (horse chestnut) [24]–[27]. The botanical origin of resin is important because propolis harvested from colonies in different climatic regions, and thus from different botanical sources, could vary in its antimicrobial properties. Indeed, it was shown that propolis samples from different regions do vary in their ability to inhibit *in vitro* growth of the bee pathogen, *Paenibacillus larvae* [28]. This effect is most likely due to the diversity in specialized metabolites secreted by the resinous plants available to bees in different regions; however, it seems that propolis has a general inhibitory effect on gram-positive bacteria and fungi [29]. This should be expected as the general inhibition of microorganisms is a role resins play in plant defense [19], [30].

Identifying the botanical sources of resins collected by honey bees can be challenging since resin foraging is relatively infrequent compared to pollen foraging [31], the variation in resin among and between plants is mostly unknown, and foraging can occur high in the canopy of trees. The botanical sources of propolis remain a mystery in most regions of the world, though 35 plant families with 88 genera that contain known resinous species occur in the continental U.S. [18], [32]. Traditional chemical analysis has been somewhat successful in identifying botanical sources of bee-collected resin by sampling at the colony level [33]. However, these methods are difficult to apply to generally unknown, variable, and complex substances, like resin, due to the amount of *a priori* information required. The exact identity of a characteristic signature compound must be known, chemically analyzed, and available as a standard. One or more of these requirements are often missing. Traditional analysis is also inefficient at describing biological variation among large numbers of samples, which is key to uncovering subtitle differences among complex mixtures. Colony-level sampling is also problematic because bees collect resin from more than one plant and mix them in the hive.

In order to identify the botanical sources of bee-collected resins and measure their species specific and seasonal variation without any prior knowledge of resin composition, we used metabolomic methods as a type of ‘environmental forensics’ to track resin forager behavior on the level of individual bees. We also used antimicrobial assays to explore potential differences in the derived benefits of collecting resin from some plants relative to resin from other plant species.

## Methods

### Sampling

Honey bees (*Apis mellifera lingustica*) were sampled from a single apiary located on the University of Minnesota, St. Paul campus. Resin was dissolved directly off the corbiculae of individual bees with acetonitrile. Resin extracts from each bee were diluted to 10% acetonitrile in water for analysis but were not normalized due to limited amounts of material.

Resin was sampled from wounds and buds of individual plants, dissolved in acetonitrile, and diluted to 1.5 mg/mL in 10% acetonitrile for analysis. Multiple wounds were sampled, if available, while six buds per individual plant were sampled.

### Data collection

**Figure 2:**
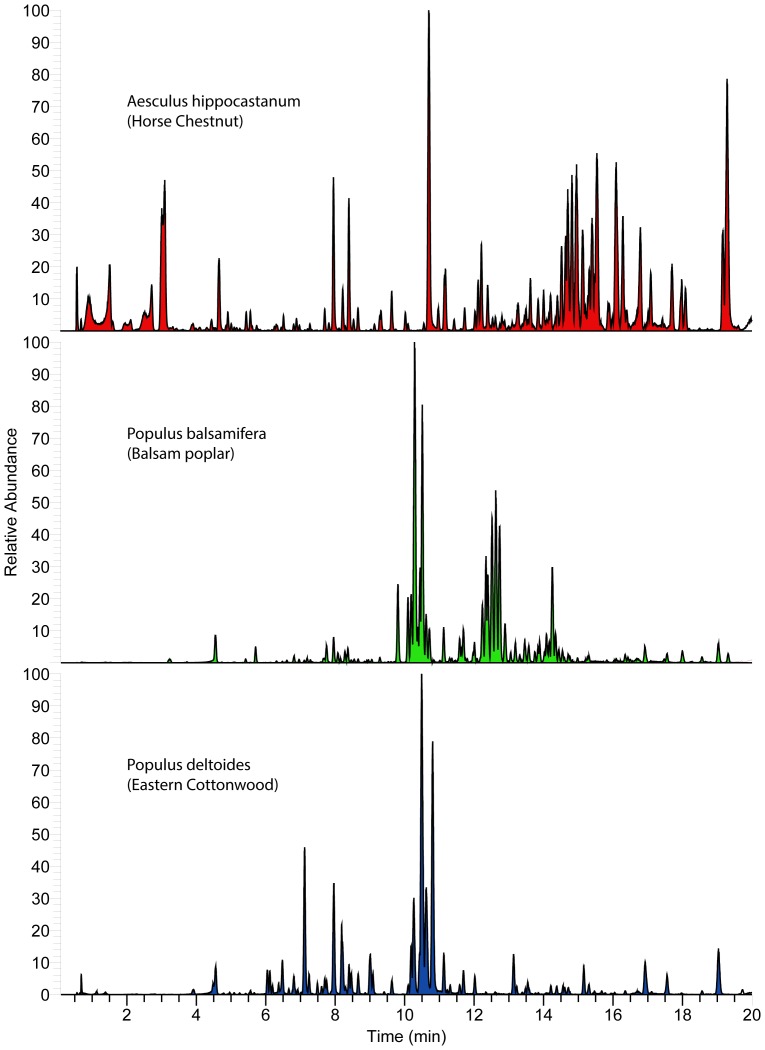
Resin metabolite diversity of studied angiosperms. Base-peak negative-ion chromatogram (“fingerprints”) of resin collected from individual *Populus spp*. (poplar) and *Aesculus hippocastanum* (horse chestnut) within 2 miles of the study apiary.

**Figure 3:**
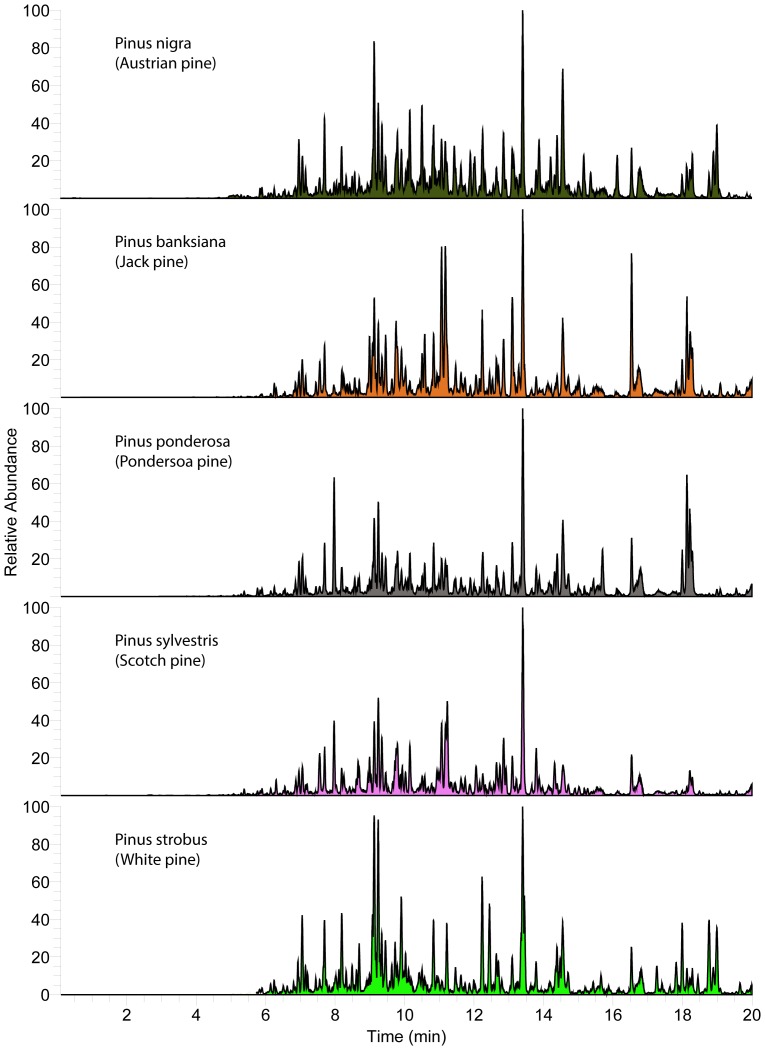
Resin metabolite diversity of studied *Pinus sp*. (Pine). Base-peak negative-ion chromatogram (“fingerprints”) of resin collected from individual *Pinus spp*. within 2 miles of the study apiary.

**Figure 4:**
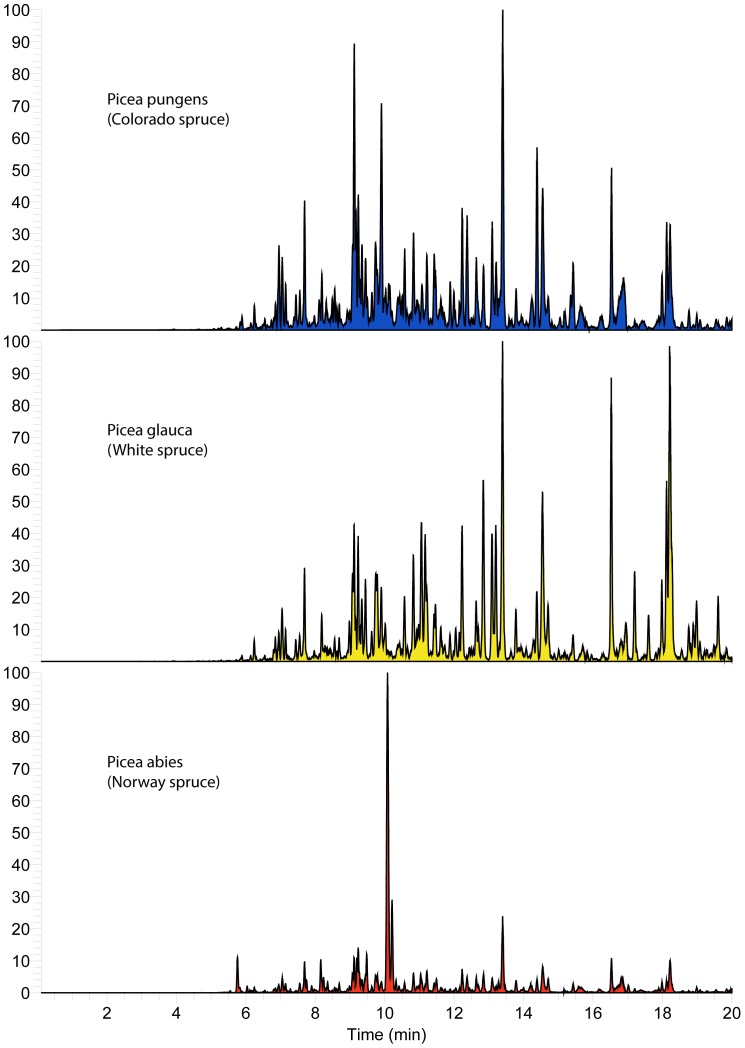
Resin metabolite diversity of studied *Picea sp*. (Spruce). Base-peak negative-ion chromatogram (“fingerprints”) of resin collected from individual *Picea spp*. within 2 miles of the study apiary.

**Figure 5:**
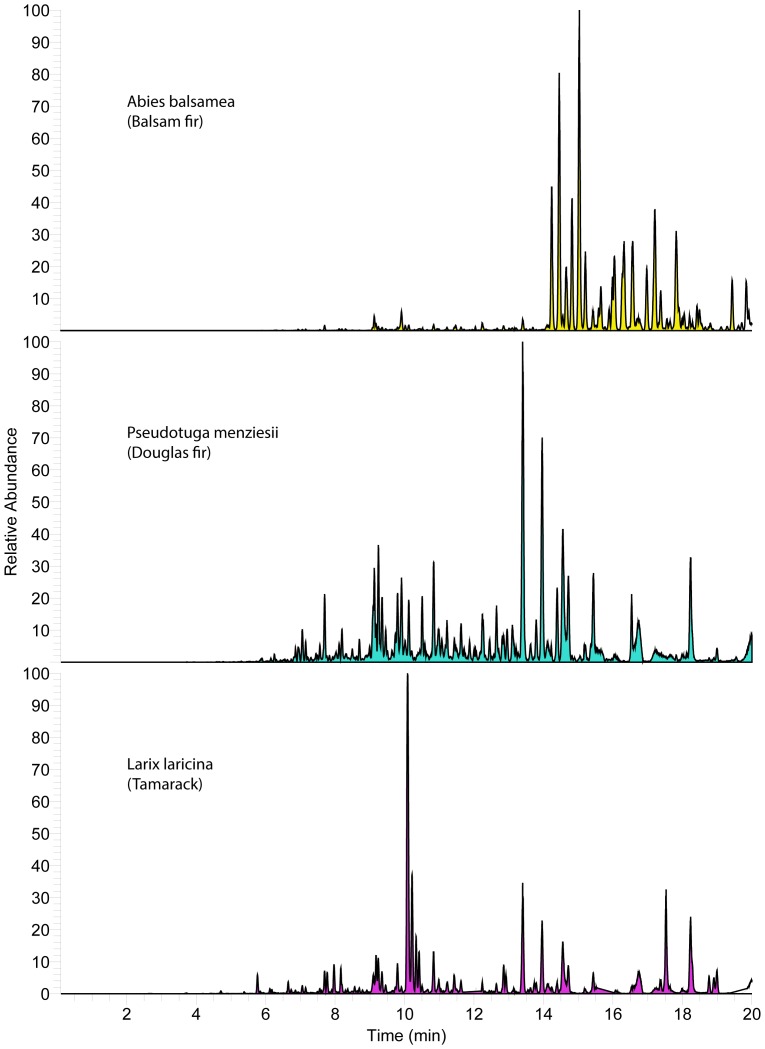
Resin metabolite diversity of other studied conifers. Base-peak negative-ion chromatogram (“fingerprints”) of resin collected from other conifers within 2 miles of the study apiary.

**Figure 6:**
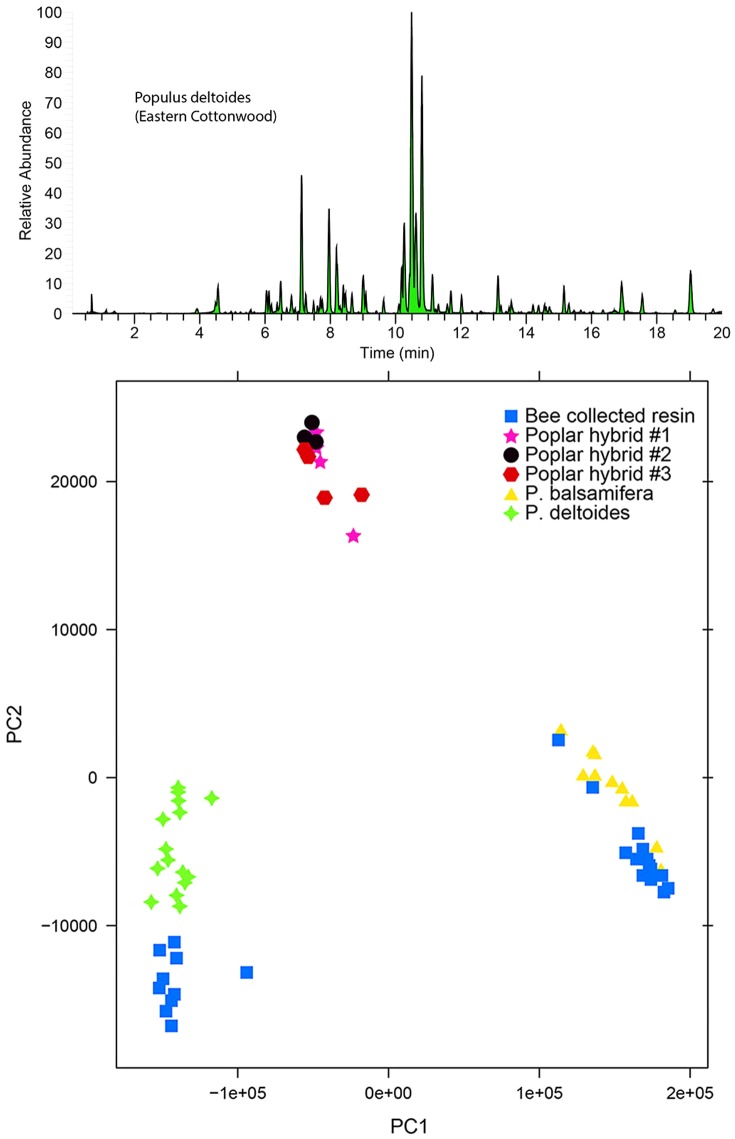
Honey bees collect resin from *P. balsamifera* and *P. deltoides*. (Top) Example of a *P. deltoides* resin metabolite ‘fingerprint’. (Bottom) PCA scores plot of resin ‘fingerprints’ from individual resin-producing plants and honey bee resin foragers. Points represent the spectral composition of a biological sample. Points that are closer together have more spectral peaks in common than with points that are farther apart. 54.35% of the total variation in the data set is shown. Hybrid poplars were sampled once in June, while *P. deltoides* (Eastern cottonwood) and *P. balsamifera* (balsam poplar) once in June and once in July. N = 25 for resin from foraging bees, N = 11 for *P. balsamifera*, N = 16 for *P. deltoides* (9 from near Jamestown, ND), and N = 5 for each poplar hybrid population.

Spectral data for Fig. 2–5 were generated using HPLC (UltiMate 3000, Thermo-Fisher Scientific, Waltham, MA) coupled to Fourier transform MS (Q-Exactive, Thermo) operated at 17,500 resolution in full scan, (−) ionization mode. Gradient: water-acetonitrile, column: Zorbax XDB C_18_ (Agilent Technologies, Santa Clara, CA), 2.1×100 mm, 1.8 µm particle size, flow rate: 350 µL/min. Metabolic fingerprints in Fig. 3, 4, and 5 were generated using UPLC (Acquity LC, Waters, Milford, MA) coupled to time-of-flight (TOF) MS (Waters LCT Premier XE) in both (+) and (−) ionization mode. Gradient: water-acetonitrile, column: Waters BEH C_18_, 1.0×100 mm, 1.8 µm particle size, flow rate: 130 µL/min. Metabolic fingerprints in Fig. 6. were generated using UPLC (Waters Acuity) coupled to TOF-MS (G2 Synapt, Waters) in both (+) and (−) ion mode. Gradient: water-acetonitrile, column: Zorbax Eclipse XDB C_18_ (Agilent), 2.1×100 mm, 1.8 µm particle size, flow rate: 350 µL/min.

### Data analyses

All data analyses were performed using a custom R script developed in our laboratory (Fig. S1). Automated peak discovery in raw MS data was preformed with the R package XCMS [34], [35] using the *centWave* algorithm. Parameters used: ppm = 10, peakwidth = c(5,50), fitgauss  =  TRUE, verbose.columns  =  TRUE. Peaks were grouped across samples using XCMS's *nearest* algorithm. Parameters used: mzCheck  = 2, rtCheck  = 5. Positive and negative ion mode peaks were combined into a single matrix for statistical analysis. A quality control sample containing equal amounts of each biological sample was run every ten samples, and spectral peaks that did not appear in all technical replicates of this quality control sample were eliminated from the analysis.

### Bacterial inhibition assay

*P. larvae* (NRRL # B2605, USDA Agricultural Research Service culture collection) was grown in brain/heart infusion broth (Difco) fortified with 1 mg/L thiamine HCl. Growth inhibition was measured spectrophotometrically (Specta Max 190, Molecular Devices), using a 96 well-plate growth assay, as the percent OD_600 nm_ of treated wells relative to untreated control wells after six hours of incubation and shaking at 37°C.

## Results

### Resin metabolite diversity available to bees

To discern what options bees have for foraging targets, the resinous plant diversity within common foraging range (3.2 km) of our St. Paul, Minnesota, campus apiaries was determined. The dominant resin-producing species in the area was *Populus deltoides* (eastern cottonwood), but *Pinus spp*. (pine) and *Picea spp*. (spruce) were also common. In addition, there were small stands of *Populus balsamifera* (balsam poplar) and hybrid poplars of unknown parentage, and scattered *Abies balsamea* (balsam fir), *Pseudotsuga menziesii* (Douglas fir), *Larix laricina* (tamarack larch), and *Aesculus hippocastanum* (horse chestnut) in the study area. *Populus tremuloides* (American aspen), *Populus grandentata* (big tooth aspen), and *Salix sp.* (willow) occurred at least once, and may be resinous in other locations [26], but were not obviously resinous at the time of sampling. It is important to note that all species were mostly interspersed among each other in the study area, though groups of *P. deltoides* and *P. balsamifera*, and individual *Pinus strobes, Pinus syvestris,* and *A. balsamea* were closest in proximity to the apiary. Sampled resins were compositionally complex with both qualitative similarities and differences, especially between genra (Fig. 2–5).

It is not known why bees forage specific resins in the field. To explore the possibility of antimicrobial activity as a criterion for resin preference, the *in vitro* activity of local resins against *Paenibacillus larvae*, a bee brood pathogen, was measured. Resin from different species varied in their ability to inhibit *P. larvae* (Table 1), with resin from *P. gluaca* being the most inhibitory, achieving complete growth inhibition at 0.05 mg/mL. *A. hippocastanum* and *P. sylvestris* resin did not completely inhibit *P. larvae* growth within the experimental concentration range, though their resins did show some inhibition of growth (data not shown).

**Table 1:** Inhibition of pathogen growth by local resins.

Resin Source	Complete growth inhibition
*Picea glauca*	0.05 mg/mL
*Larix laricina*	0.06 mg/mL
*Pinus banksiana*	0.06 mg/mL
*Pinus ponderosa*	0.06 mg/mL
*Populus balsamifera*	0.075 mg/mL
*Picea abies*	0.1 mg/mL
*Pinus nigra*	0.1 mg/mL
*Abies balsamea*	0.125 mg/mL
*Picea pungens*	0.125 mg/ml
*Pinus strobus*	0.125 mg/mL
*Pseudotsuga menziesii*	0.175 mg/mL
*Populus deltoides*	0.175 mg/mL
*Aesculus hippocastanum*	>0.175 mg/mL
*Pinus sylvestris*	>0.175 mg/mL

Table describes the concentration at which the bee pathogen, *Paenibacillus larvae*, was inhibited by resin collected from local plants in a spectrophotometric growth assay completely (≤ 1% OD_600_ of untreated controls).

### Using metabolomic forensics to reveal the botanical sources of resin

Twenty six individual resin foraging bees, typically carrying ≤ 5 mg of resin, were captured returning to two colonies over three sampling events in July. Captured bees collected dark red and bright yellow resins, which match the visual description of resin from *P. balsamifera* (red), and *P. deltoides* (yellow), or hybrid poplars of unknown variety (yellow) occurring in the area. *P. deltoides*, *P. balsamifera*, and hybrid poplars occurring within two miles of the experimental apiary were sampled in June and July by washing resinous buds with acetonitrile. Resin was also collected from *P. deltoides* near Jamestown, ND, in July to test for regional variation. It is unclear how the environment impacts the expression of resin metabolites in *Populus spp*., though it has been reported that increased light intensity does increase leaf resin accumulation in tropical *Hymenaea* and *Copaifera* species without effecting resin composition [34]. Jamestown, ND is ∼510 km northwest of the St. Paul study site, has a slightly drier and cooler climate, and a significantly different landscape (urban vs. prairie/wetland).

Metabolite “fingerprints” of resin samples collected from bees and plants were acquired by RPHPLC time-of-flight (TOF) mass spectrometry in both positive and negative ion modes. A quality control sample was created by pooling equal amounts of all biological samples (thus theoretically containing all peaks that could occur in the resulting dataset) and injected after every 10 samples throughout the analytical run to act as a reference for automated peak detection. Spectral peaks were discovered in the analytical data with the R package XCMS [35], [36] using the *centWave* algorithm and matched across different samples using the *nearest* algorithm. Only peaks discovered in all technical replicates of the quality control sample were included in our analysis (313 spectral peaks), to ensure that only high confidence peaks would be used in the statistical analysis. This data analysis strategy was directed toward the discovery of unique and exclusive metabolites among samples and not focused on differences in concentrations or proportions. Principal components analysis (PCA) was used to summarize metabolite patterns in the data (Fig. 6). Sensitivity testing showed that the grouping pattern of the PCA scores plot was not greatly affected by performing different scaling transformations (log_(X)_) of the peak intensity data, which influences the importance of peak intensity differences relative to the presence/absence of peaks between samples in the analysis.

Bee collected resin and plant collected resin group together in PCA space, with 10 bees foraging from *P. deltoides*, 15 bees foraging from *P. balsamifera*, and no bees foraging from any of the hybrid poplar populations (Fig. 6). One bee foraged from an unknown source that did not match the patterns of any previously sampled plant species, and was eliminated from the analysis (data not shown). The regional variation between *P. deltoides* resin collected in Jamestown, ND vs. resin collected in St. Paul, MN was negligible.

### Seasonal variation in P. deltoides and P. balsamifera resin

Propolis composition has been reported to change seasonally in some regions [37]. This could be due to changes in bee foraging behavior, where bees change their preference for some resin plants over others over the course of the season. Alternatively, seasonal changes in propolis composition could be due to seasonal changes in resin availability or composition, such as changes in plant resin flow or changes in plant specialized metabolism.

**Figure 7:**
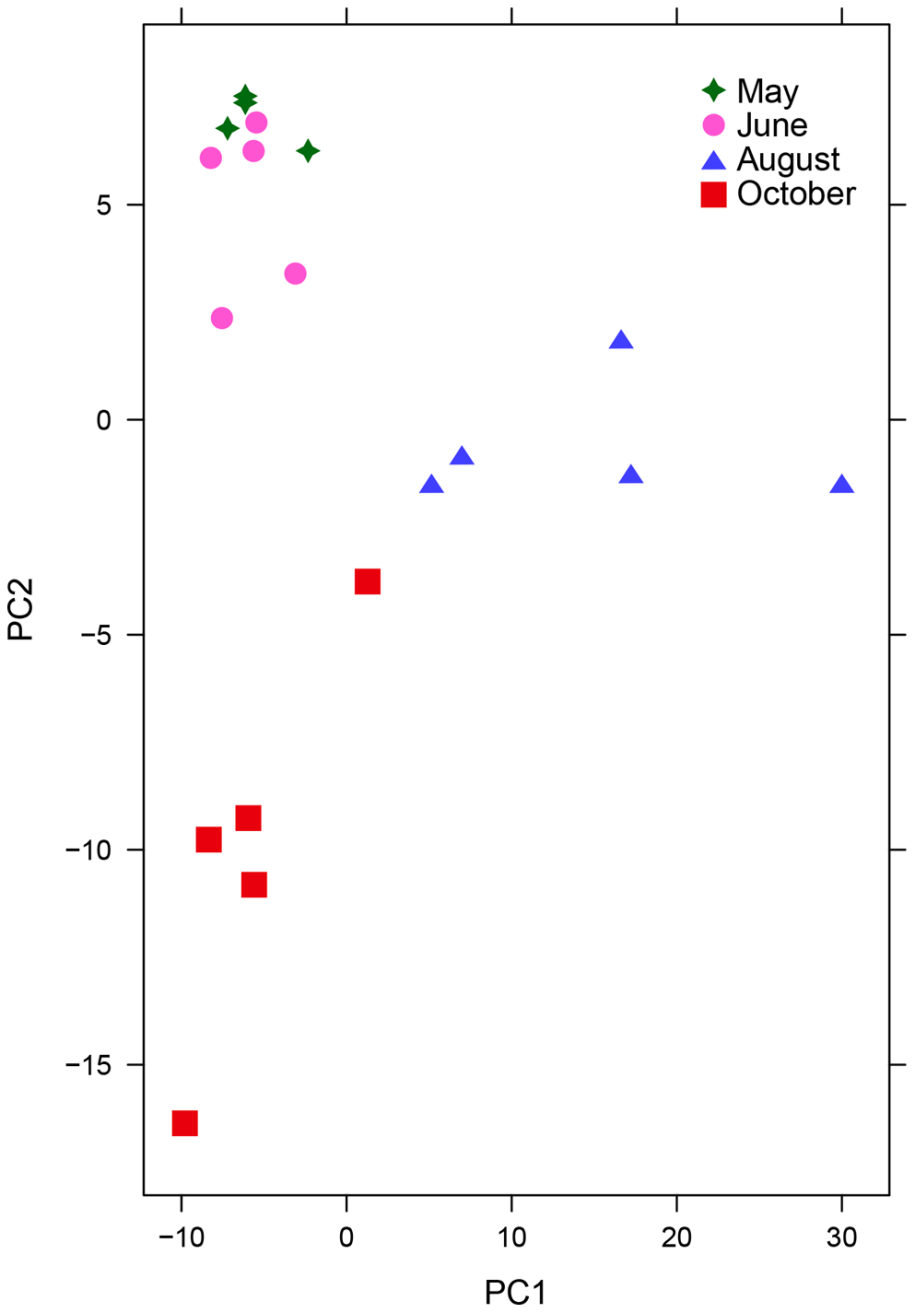
Seasonal variation in *P. balsamifera*. PCA scores plot of resin ‘fingerprints’ from individual *P. balsamifera* plants collected throughout the growing season. 43.99% of the total variation in the data set is shown. N = 4 individuals in May, N = 5 individuals in June, August, and October.

We sampled *P. deltoides* and *P. balsamifera* around the experimental apiary throughout the 2011 resin collection season (May, June, July, August, and October) to test if detectable changes in resin composition occurred. Data acquisition and analysis were preformed as described in the previous section. Fig. 7 shows a gradual shift in *P. balsamifera* resin composition by month. The data agree with our visual observations that *P. balsamifera* resin changes from yellow/orange in the active growing season to dark red when buds begin to set for the winter. Out of 382 *P. balsamifera* spectral peaks, only one verified peak was exclusively found in all October samples (*m/z* = 483.409, RT = 23.53 min., positive ion mode). 13 other verified peaks were identified as possible late season indicators (Table 2).

**Table 2:** Spectral peaks representing late-season indicators in *P. balsamifera* and *P. deltoides*.

Mass (*m/z*)	Rt (min.)	Ion Mode	Appearance (by month)
***P. balsamifera***			
483.409	23.53	+	5/5 Oct
353.099	11.07	−	4/5 Oct
621.271	14.57	−	4/5 Oct
595.205	14.85	−	4/5 Oct
491.288	14.57	+	1/5 Aug, 5/5 Oct
475.247	14.57	−	1/5 Aug, 5/5 Oct
531.279	14.57	+	1/5 Aug, 4/5 Oct
461.272	14.83	+	1/5 Aug, 4/5 Oct
625.212	14.57	−	1/5 Aug, 4/5 Oct
653.208	14.57	−	1/5 Aug, 4/5 Oct
519.276	15.77	−	1/5 Aug, 4/5 Oct
537.273	16.27	−	1/5 Aug, 4/5 Oct
521.292	15.98	−	3/5 Aug, 5/5 Oct
373.291	19.6	−	1/5 June, 5/5 Oct
***P. deltoides***			
339.220	13.15	−	5/5 Oct
295.220	12.52	−	5/5 Oct
293.220	13.15	−	4/5 Oct
295.239	13.13	+	4/5 Oct
503.401	18.35	+	1/4 Aug, 5/5 Oct
519.356	16.25	−	1/4 Aug, 5/5 Oct
525.384	18.35	+	2/4 Aug, 5/5 Oct

Table arranged by peak appearance in a given sample group. The number of samples within a month group in which each peak appears is indicated in the last column. Retention time was rounded to the nearest 0.1 min. Mass accuracy was 5–10 ppm. Rt = retention time.

**Figure 8:**
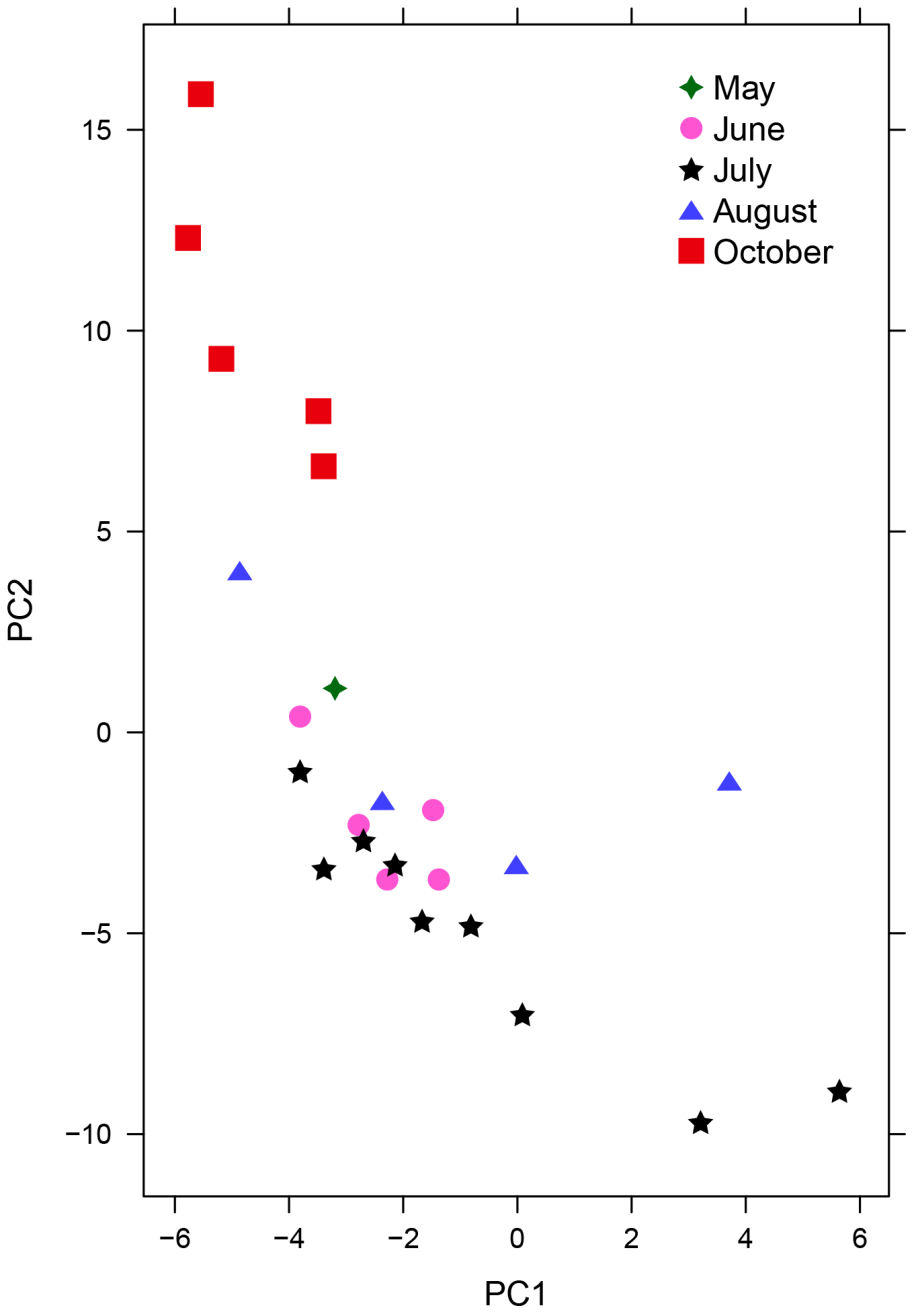
Seasonal variation in *P. deltoides*. PCA scores plot of resin ‘fingerprints’ from *P. deltoides* plants collected throughout the growing season. 40.86% of the total variation in the data set is shown. N = 2 individuals in May, N = 5 individuals in June, N = 9 individuals in July (from near Jamestown, ND), N = 5 individuals in August, N = 5 individuals in October.

The seasonal changes observed in *P. deltoides* resin were simpler than those observed for *P. balsamifera* resin. Two distinct groups formed, resin collected in October vs. resin collected in all other months (Fig. 8). Out of 352 *P. deltoides* spectral peaks, two verified peaks were found exclusively in all October samples (*m/z* = 339.220, RT = 13.15 min.; *m/z* = 295.220, 12.62 min., both negative ion mode). Seven other verified peaks were identified as possible late season indicators (Table 2).

### Difference in resin metabolites and biological activity among *Populus spp*

The genus *Populus* is widely regarded as a preferential source of resin for honey bees in temperate climates [28], [32]. Clearly, this is true at the apiary examined in this study (Fig. 6), although it is not known why *Populus* is preferable to other resin producers or if there are preferences among *Populus spp*. Studies focused on phenolic compounds extractable with diethyl ether and analyzed as their trimethylsilyl derivatives have shown that resins from North American *Populus spp*. can have different compositions [38]–[40].

**Figure 9:**
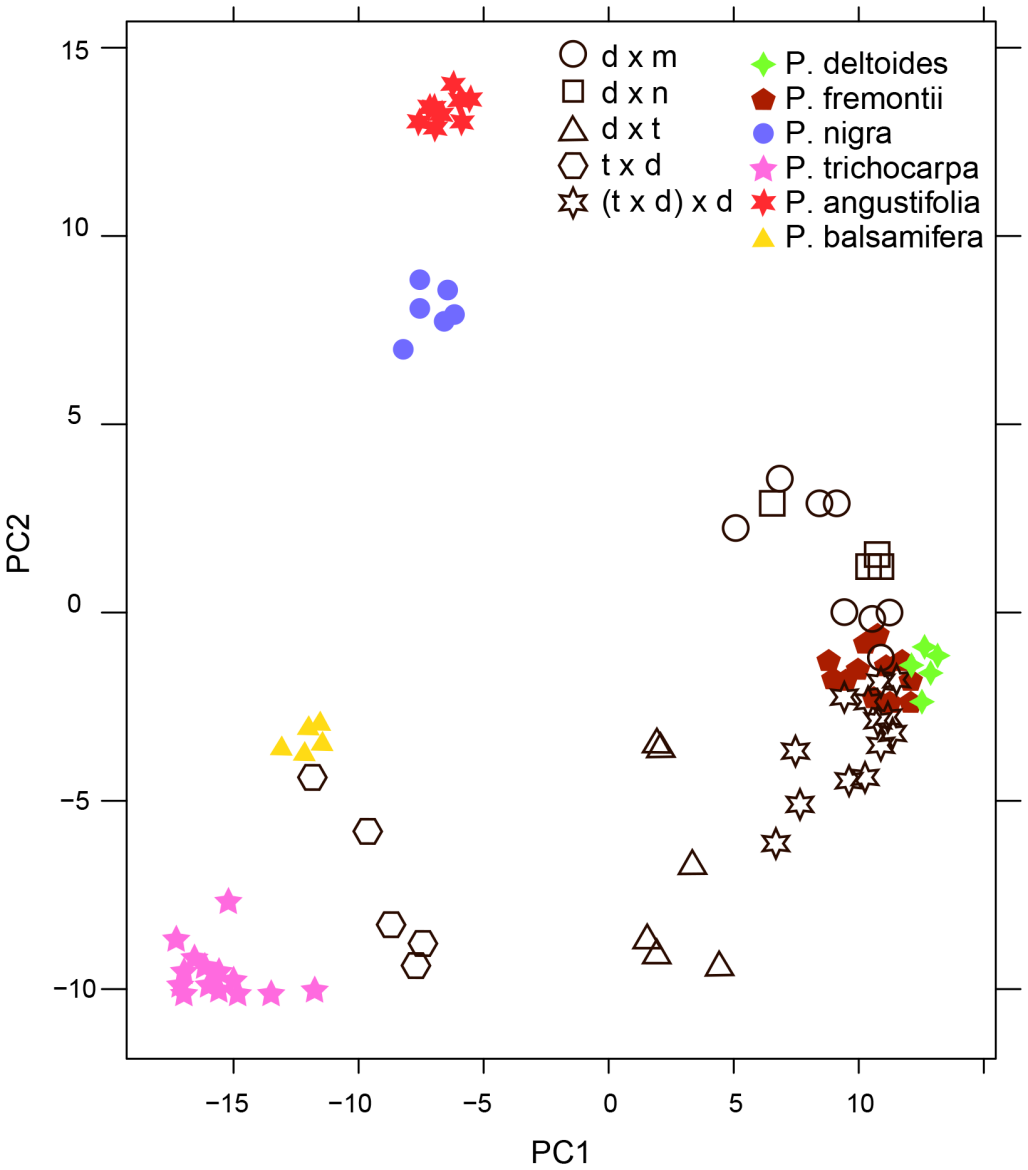
Compositional differences in *Populus spp.* resin. PCA scores plot of resin ‘fingerprints’ from 11 different *Populus spp.* and hybrids grown under greenhouse conditions. Pure species are indicated by closed shapes, while hybrids are indicated by open shapes. 49.11% of the total variation in the data set is shown. d x m  =  *P. deltoides x maximowiczii* (N = 8), d x n = *P. deltoides x nigra* (N = 4), d x t = *P. deltoides x trichocapra* (N = 6), t x d = *P. trichocarpa x deltoides* (N = 5), (t x d) x d = *P. (trichocarpa x deltoides) x deltoides* (N = 14). N = 6 for *P. deltoides* and *P. nigra*, N = 12 for *P. fremontii*, N = 14 for *P. trichocarpa*, N = 18 for *P. angustifolia*, N = 5 for *P. balsamifera*.

To test the diversity of metabolite composition and bee-relevant antimicrobial activities of North American poplar resins, 11 species and hybrids were propagated via hardwood cuttings in the greenhouse for analyses (*P. balsamifera*, *P. angustifolia*, *P. trichocarpa*, *P. nigra*, *P. deltoides*, *P. fremontii*, *deltoides* x *trichocarpa*, *trichocarpa* x *deltoides*, *deltoides* x *nigra*, *deltoides* x *maximowiczii*, (*deltoides* x *trichocarpa*) x *trichocarpa*). Resin was harvested and analyzed as described previously. Only one *P. deltoides* cutting survived, so the analysis was supplemented with 5 samples of *P. deltoides* resin collected in the study area used previously. Fig. 9 summarizes the compositional relatedness found among the different species/hybrids. Of 344 spectral peaks, several exclusive peaks were characteristic of *P. trichocarpa*, *P. angustifolia*, or *P. deltoides x maximowiczii* respectively (Table 3), and each species/hybrid had a unique combination of non-exclusive spectral peaks that appeared in all resin samples from a given species/hybrid. Most species/hybrids did not have any exclusively characteristic spectral peaks, however many peaks were found to be characteristic of terminal phylogenic nodes [41] within *Populus* (*P. deltoides*/P. *fremontii*, *P. angustifolia*, *P. balsamifera*/*P. trichocarpa* - Table 3).

**Table 3:** Spectral markers of terminal taxonomic nodes in *Populus*.

Mass (*m/z*)	Rt (min)	Ion Mode	Appearance (by species)
**Taxonomic Section – ** ***Aigeiros***			
291.0634	6.33	+	6/6 Pd, 12/12 Pf
563.1664	6.83	+	6/6 Pd, 12/12 Pf
395.1100	6.95	+	6/6 Pd, 12/12 Pf
307.0584	7.77	+	6/6 Pd, 12/12 Pf
241.0866	7.77	+	6/6 Pd, 12/12 Pf
269.0825	7.77	+	6/6 Pd, 12/12 Pf
329.1025	7.77	+	6/6 Pd, 12/12 Pf
357.1330	10.1	+	6/6 Pd, 12/12 Pf
379.1147	10.1	+	6/6 Pd, 12/12 Pf
273.0770	12.1	+	6/6 Pd, 12/12 Pf
267.0644	6.33	−	6/6 Pd, 12/12 Pf
327.0863	7.8	−	6/6 Pd, 12/12 Pf
313.0737	9.6	−	6/6 Pd, 12/12 Pf
**Taxonomic Section - ** ***Tacamahaca***			
363.1214	9.4	+	18/18 Pa
339.1215	9.4	−	18/18 Pa
121.0654	9.1	+	5/5 Pb, 14/14 Pt
301.1074	10.85	+	5/5 Pb, 14/14 Pt
303.1239	11.3	+	5/5 Pb, 14/14 Pt
421.1654	11.4	+	5/5 Pb, 14/14 Pt
121.0652	11.3	+	5/5 Pb, 14/14 Pt
301.1083	11.4	+	5/5 Pb, 14/14 Pt
553.2219	12.4	+	5/5 Pb, 14/14 Pt
301.1086	13.0	+	5/5 Pb, 14/14 Pt
285.1126	13.4	+	5/5 Pb, 14/14 Pt
553.2221	13.4	+	5/5 Pb, 14/14 Pt
315.1225	15.2	+	5/5 Pb, 14/14 Pt
405.1182	4.3	−	5/5 Pb, 14/14 Pt
287.0912	9.1	−	5/5 Pb, 14/14 Pt
389.1389	11.6	−	5/5 Pb, 14/14 Pt
551.2079	12.4	−	5/5 Pb, 14/14 Pt
521.1958	12.5	−	5/5 Pb, 14/14 Pt
433.1644	13.1	−	5/5 Pb, 14/14 Pt
404.1544	13.4	−	5/5 Pb, 14/14 Pt
535.2126	15.3	−	5/5 Pb, 14/14 Pt
437.2103	6.4	+	14/14 Pt
551.1533	6.7	+	14/14 Pt
161.1306	9.0	+	14/14 Pt
135.0412	2.8	−	14/14 Pt
**Taxonomic Section - ** ***Aigeiros/Tacamahaca***			
209.0802	9.1	−	8/8 dm

Table arranged by peak appearance in a given species group. The number of samples within a species group in which each peak appears is indicated in the last column. Retention time was rounded to the nearest 0.1 min. Pd  =  *P. deltoides*, Pf  =  *P. fremontii*, Pa  =  *P. angustifolia*, Pb  =  *P. balsamifera*, Pt  =  *P. trichocarpa*, dm  =  *P. deltoides x maximowiczii*. Mass accuracy was 2 ppm. Rt  =  retention time.

**Figure 10:**
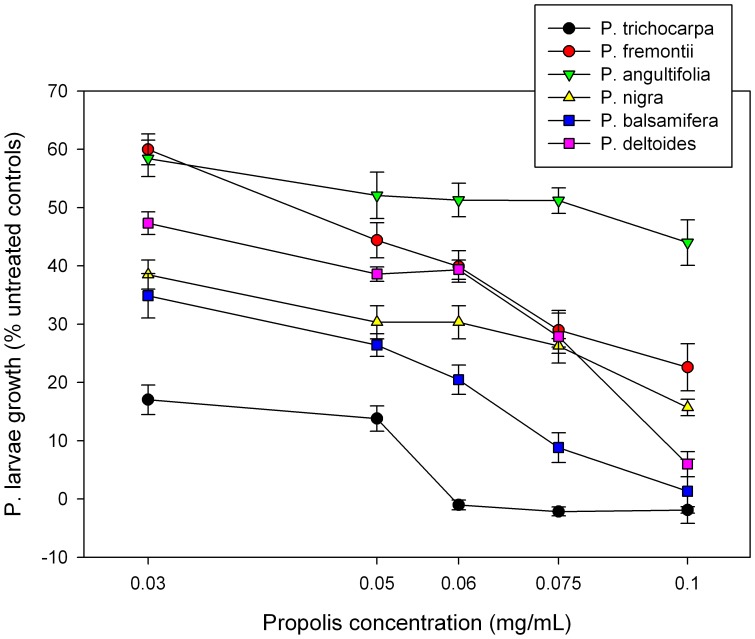
Inhibition of *Paenibacillus larvae* by *Populus spp.* resin. Semi-log plot of the antimicrobial activity of resin from six representative *Populus spp*. individuals against *P. larvae*, a brood pathogen of honey bees. Antimicrobial activity was evaluated spectrophotmetrically at OD_600_ relative to untreated controls. N = 8 replicates per sample per concentration of resin.

As the range of many abundant North American *Populus spp.* overlap, bees are commonly presented with a choice between closely related resin producing plants. To test the diversity in potential benefit of North American poplar resins to bees, resin extracted for analytical analysis was also tested for antimicrobial activity against *P. larvae*. Fig. 10 clearly shows differences in antimicrobial activity among the different species with *P. trichocarpa* being the most and *P. angustifolia* the least inhibitory.

## Discussion

Honey bees in the study region have many resin sources to choose from that are diverse in metabolite content and antimicrobial activity (Fig. 2–5), and it appears they favor some plants over others (Fig. 6). Strong resin plant preference has been shown in stingless bees (Tribe: Meliponini) [42]–[43] as well as a slight male over female *B. dracunculifolia* preference (∼10%) by honey bees in Brazil [44].

To our knowledge, this is the first example of using chemical analysis to track individual resin forager behavior. Importantly, these methods were sensitive enough to track resin foraging behavior by analyzing the resin from a single leg of an individual bee. Metabolomic forensics confirmed that honey bees collected resin from *P. balsamifera* and *P. deltoides* (Fig. 6), but not from the numerous hybrid poplars located within common foraging range. We confirmed that honey bees have a high fidelity to a single botanical source of resin during a single foraging trip, even when multiple closely related species occur in very close proximity that are also active foraging targets (Fig. 6).

Regional environment had little effect on *P. deltoides* resin composition when comparing resin sampled near Jamestown, ND and in St. Paul, MN (Fig. 6), while season had only a subtitle effect on overall resin composition, mostly among resins collected in October compared to all earlier months (Fig. 7 & 8). The relevance of these subtle compositional changes in relation to bee activity is uncertain, as all foraging stops by mid-September in the study region. At least in this study area, large changes in propolis composition by season would probably be due to changes in resin availability or forager preference, and not by gross shifts in specialized plant metabolism.

Resin sampled from greenhouse grown North American poplars showed that some metabolites were unique to taxonomic nodes, with resin from closely related species being surprisingly different (Table 3, Fig. 9). Hybridization did not seem to produce novel metabolites. *P. deltoides x trichocarpa* and *P. trichocarpa x deltoides* resins had different intermediate compositions compared to their parental species (Fig. 9), which may indicate some maternal effects on resin metabolism. Due to the clear appearance of taxonomic node-specific metabolites, future work could provide insight into the evolution of specialized metabolism in the *Populus* genus. Overall, this metabolomics approach provided a powerful method to discriminate among patterns of resin metabolites from closely related species and hybrids, while also identifying specific metabolites that were characteristic signatures among species/hybrid groups.

Collected resins varied in antimicrobial activity against the highly infectious brood pathogen, *P. larvae* (Table 1, Fig. 6). Although the study colonies were not infected with this pathogen, the data suggest that availability, proximity, and perhaps toxicity may play roles in the selection of resins by bees. *P. deltoides* and *P. balsamifera* were targets of resin foraging, and also the closest abundant species to our experimental apiary. North American poplars differentially inhibited the growth of *P. larvae* and Fig. 10 shows that even among plant species in the same genus, a bee's choice of resin could have profound consequences for their ability to reduce the overall microbe load within the nest cavity and prevent or fight off disease. *P. balsamifera* resin was more strongly inhibitory than *P. deltoides* resin, but there was no obvious preference for the more inhibitory resin among captured resin foragers (Fig. 6 & Fig. 9). Future studies with deeper sampling might uncover aspects of preferences not obvious with the sample size used in this study.

As many North American poplars commonly co-occur in the same environment (*P. deltoides* and *P. balsamifera* in Minnesota, for example), bees often have to choose between closely related resin sources. It is not well understood how bees locate preferential resin sources, but resin foraging frequency does increase in several bee species under certain conditions. Stingless bees increase resin foraging in response to ant attacks, while honey bees increase resin foraging when intentionally exposed to the larval fungal pathogen *Ascosphaera apis*, the cause of chalkbrood [31]. It would be informative to determine if bees change resin preference, along with frequency, during these events. Resin foraging may also be a tactile response to crevices and rough textures within the hive [42], [45], and roughing the inside surface of standard bee boxes might encourage the deposition of more resin.

Chemical analysis is a highly informative alternative to observational studies of resin foraging, as direct observation of resin foraging is difficult and prone to error. Metabolomic methods uniquely allow for the analysis of many samples without targeting specific signature metabolites. We were able to observe patterns in >300 peaks in over 100 samples and summarize their differences during a single analysis (Fig. 9), which could not be accomplished with traditional analyses. Many of the peak differences detected were not obvious and would likely have been missed with only visual inspection of the raw LC-MS data.

We seek to understand the botanical sources and biological activities of resins in the field and how resin foraging behavior changes in response to environmental factors, such as infection and other biological stresses. If we can discover plants with preferable and more antimicrobial resins in different regions, it should be possible to better create environments that promote bee health by supporting behaviors and managerial strategies that lead to natural disease resistance.

## Supporting Information

Figure S1 **R script for comparative metabolomic analysis.** This script is a conservative, open source data analysis procedure for metabolomics experiments that use a ‘composite’ quality control sample, as described in the results subsection “Honey bees collect resin from balsam poplar and eastern cottonwood.”
(DOC)

## References

[1] Schmid-Hempel P (1998) Parasites in social insects. Princeton:Princeton University Press.

[2] vanEngelsdorp D, Meixner MD (2010) A historical review of managed honey bee population in Europe and the United States and the factors that may affect them. . J. Invertebr. Path 103: S80–S95. 19909973 10.1016/j.jip.2009.06.011

[3] vanEngelsdorp D, Dewey C, Hayes J, Underwood R, Henson M, et al (2012) A nation survey of managed honey bee 2010–2011 winter colony losses in the USA: Results from the Bee Informed Partnership. . J. Apic. Res 51(1): 115–124.

[4] Cox-Foster DL, Conlan S, Holmes EC, Palacios G, Evans JD, et al (2007) A metagenomic survey of microbes in honey bee colony collapse disorder. Science 318: 283–287. 17823314 10.1126/science.1146498

[5] vanEngelsdorp D, Evans JD, Saegerman C, Mullin C, Haubruge E, et al (2009) Colony collapse disorder: A descriptive study. PloS ONE 4: e6481 10.1371/journal.pone.0006481 19649264 PMC2715894

[6] van Dooremalen C, Gerristsen L, Cornelissen B, van der Steen JJM, van Langevelde F, et al (2012) Winter survival of individual honey bees and honey bee colonies depends on level of *Varroa destructor* infestation. PloS ONE 7(4): e36285 10.1371/journal.pone.0036285 22558421 PMC3338694

[7] Johnson RM, Ellis MD, Mullin CA, Frazier M (2010) Pesticides and bee toxicity – USA. Apidologie 41: 312–331.

[8] Alaux C, Ducloz F, Crauser D, Le Conte Y (2010) Diet effects on honeybee immunocompetence. Biol. Lett. 6: 562–565. 10.1098/rsbl.2009.0986 PMC2936196 20089536

[9] Johnson R (2010) Honey bee colony collapse disorder. Congressional Research Service

[10] Evans JD, Aronstein K, Chen YP, Hetru C, Imler JL, et al (2006) Immune pathways and defence mechanisms in honey bees *Apis mellifera*. . Insect Mol. Biol 15(5): 645–656. 17069638 10.1111/j.1365-2583.2006.00682.x PMC1847501

[11] Cremer S, Armitage S, Schmid-Hempel P (2007) Social Immunity. Curr. Biol. 17: R693–R702. 10.1016/j.cub.2007.06.008 17714663

[12] Wilson-Rich N, Spivak M, Fefferman NH, Starks PT (2009) Genetic, individual, and group facilitation of disease resistance in insect societies. . Annu. Rev. Entomol 54: 405–423. 18793100 10.1146/annurev.ento.53.103106.093301

[13] Evans JD, Spivak M (2009) Socialized medicine: Individual and communal disease barriers in honey bees. J. Invertebr. Path. 103: S62–S72. 10.1016/j.jip.2009.06.019 19909975

[14] Gilliam M, Taber III S, Lorenz BJ, Prest DB (1988) Factors affecting development of chalkbrood disease in colonies of honey bees, *Apis mellifera*, fed pollen contaminated with *Ascosphaera apis*. . J. Invertebr. Path 52: 314–325.

[15] Spivak M, Reuter GS (2001) Resistance to American foulbrood disease by honey bee colonies *Apis mellifera* bred for hygienic behavior. Apidologie 32: 555–565.

[16] Simone M, Evans JD, Spivak M (2009) Resin collection and social immunity in honey bees. Evolution 63: 3016–3022. 19619221 10.1111/j.1558-5646.2009.00772.x

[17] Seely TD, Morse RA (1976) The nest of the honey bee *Apis mellifera L*. Insect. Soc. 23: 495–512.

[18] Langenheim J (2003) Plant Resins: Chemistry, Evolution, Ecology, Ethnobotany. Portland: Timber Press. 23–26 p, 477–485 p.

[19] Arrhenius SP, Langenheim JH (1983) Inhibitory effects of *Hymenaea* and *Copaifera* leaf resins on the leaf fungus, *Pestalotia subcuticularis*. Biochem. Sys. Ecol. 11(4): 361–366.

[20] Langenheim JH, Hall GD (1983) Sesquiterpene deterrence of a leaf-typing lepidopteran, *Stenoma ferrocanella*, on *Hymenaea stigonocarpa* in central Brazil. Biochem. Sys. Ecol. 11(1): 29–36.

[21] Witham TG (1983) Host manipulation of parasites: within plant variation as a defense against rapidly evolving pests. In Variable Plants and Herbivores in Natural and Managed Systems, New York: Academic Press. 15–41 p.

[22] Chapuist M, Oppliger A, Magliano P, Christe P (2007) Wood ants use resin to protect themselves against pathogens. . Proc. R. Soc. London. B 247: 2013–1017. 10.1098/rspb.2007.0531 PMC2275180 17535794

[23] Mennerat A, Perret P, Bourgault P, Blondel J, Gimenez O, et al (2009) Aromatic plants in the nest of blue tits: positive effects on nestlings. . Anim. Behav 7: 569–574.

[24] Ghisalberti EL (1976) Propolis: a review. Bee World 60: 59–84.

[25] Alfonsus EC (1933) Some sources of propolis. Glean. Bee Cult. 61: 92–93.

[26] Crane E (1990) Bees and Beekeeping: Science, Practice, and World Resources. Ithaca: Cornell University Press. 367–370 p.

[27] Bankova VS, de Castro SL, Marcucci MC (2000) Propolis: recent advances in chemistry and plant origin. Apidologie 31: 3–15.

[28] Bastos EMAF, Simone M, Jorge DM, Soares AEE, Spivak M (2008) *In vitro* study of the antimicrobial activity of Brazilian propolis against *Paenibacillus larvae*. . J. Inverter. Path 97: 273–281. 10.1016/j.jip.2007.10.007 18054037

[29] Kujumgiev A, Tsvetkova I, Serkedjieva Y, Bankova V, Christov R, et al (1999) Antibacterial, antifungal and antiviral activity of propolis of different geographic origin. . J. Ethnopharmacol 64: 235–240. 10363838 10.1016/s0378-8741(98)00131-7

[30] Levin DA (1976) The chemical defenses of plants to pathogens and herbivores. Annu Rev Ecol Syst 7: 121–159.

[31] Simone-Finstrom MD, Spivak M (2012) Increased resin collection after parasite challenge: A case of self-medication in honey bees? PLoS One 7 3: e34601, 10.1371/journal.pone.0034601 22479650 PMC3315539

[32] USDA, NRCS National Plant Data Team (2013) USDA PLANTS Database, plants.usda.gov. Accessed 2013 Sept 16.

[33] Bankova VS, Popova M, Trusheva B (2006) Plant sources of propolis: an update from a chemist's point of view. . Nat. Prod. Commun 1: 1023–1028.

[34] Langenheim JH, Arrhenius SP, Nascimento JC (1981) Relationship of light intensity to leaf resin composition and yield in the tropical lenguminous gernera *Hymenaea* and *Copaifera*. Biochem. Syst. Ecol. 9(1): 27–37.

[35] Smith CA, Want EJ, O′Maille G, Abagyan R, Siuzdak G (2006) XCMS: Processing mass spectrometry data for metabolite profiling using nonlinear peak alignment, matching, and identification. . Anal. Chem 78: 779–787. 16448051 10.1021/ac051437y

[36] Tautenhahn R, Böttcher C, Neumann S (2008) Highly sensitive feature detection for high resolution LC/MS. BMC Bioinformatics 9 504, 10.1186/1471-2105-9-504 PMC2639432 19040729

[37] Bankova V, Boudourova-Krasteva G, Popov S, Sforcin JM, Funari C (1998) Seasonal variations of the chemical composition of Brazilian propolis. Apidologie 29: 361–367.

[38] Greenway W, Jobling J, Scaysbrook T (1987) Composition of bud exudate of Populus x interamericana clones as a guide to clonal identification. Silvae Genetica 38(1): 28–32.

[39] English S, Greenway W, Whatley FR (1991) Analysis of phenolics of *Populus trichocarpa* bud exudates by GC-MS. Phytochemistry 30(2): 531–533.

[40] English S, Greenway W, Whatley FR (1992) Analysis of phenolics in the bud exudates of *Populus deltoides*, *P. fremontii*, *P. sargentii* and *P. wislizenii* by GC-MS. Phytochemistry 31(4): 1255–1260.

[41] Hamzeh M, Dayanandan S (2004) Phylogeny of *Populus* (Salicaceae) based on nucleotide sequences of chloroplast trnT-trnF region and nuclear rDNA. . Am. J. Bot 91(9): 1398–1408. 21652373 10.3732/ajb.91.9.1398

[42] Leonhardt SD, Bluthgen N (2009) A Sticky affair: Resin collection by Bornean stingless bees. Biotropica 41(6): 730–736.

[43] Leonhardt SD, Zeilhofer S, Bluthgen N, Schmitt T (2010) Stingless bees use terpenes as olfactory cues to find resin sources. Chem. Senses 35: 603–611. 10.1093/chemse/bjq058 20534774

[44] Teixeira EW, Negri G, Meira RMSA, Message D, Salatino A (2005) Plant origin of green propolis: Bee behavior, plant anatomy and chemistry. eCAM 2(1): 85–92. 15841282 10.1093/ecam/neh055 PMC1062148

[45] Simone-Finstrom M, Gardner J, Spivak M (2010) Tactile learning in resin foraging honeybees. Behav. Ecol. Sociobiol. 64(10): 1609–1617.

