# Supplementary material for: Metabolomics Reveals the Origins of Antimicrobial Plant Resins Collected by Honey Bees
Source: PLoS One. 2013 Oct 18;8(10):e77512. doi: 10.1371/journal.pone.0077512 (PMC3799627; doi:10.1371/journal.pone.0077512)
Supplement: Figure S1 — R script for comparative metabolomic analysis. This script is a conservative, open source data analysis procedure for metabolomics experiments that use a ‘composite’ quality control sample, as described in the results subsection “Honey bees collect resin from balsam poplar and eastern cottonwood.” (DOC) [file pone.0077512.s001.doc]

**Figure S1 – R script for comparative metabolomic analysis.** This script is a conservative, open source data analysis procedure for metabolomics experiments that use a ‘composite’ quality control sample, as described in the results subsection “Honey bees collect resin from balsam poplar and eastern cottonwood.”

############################################################

## Setting up filepaths, names, and packages

## Before you start - Your active directory should contain all your data files and a directory #called 'results'

## You will need to download the following packages: ‘xcms’, ‘RANN’, and ‘lattice’

##Load dependent packages

library(xcms)

library(RANN)

library(lattice)

## about.csv is a file with information about the raw data files in the 'results' directory

## about.csv needs 4 columns: id (a unique identifier), group (sample treatment, ect), Nfile (neg #mode file path), Pfile (pos mode file path)

## Your quality control group must be labeled as “Composite” in the group column

## Set up a name and read in your 'about' file

file.about <- "results/about.csv"

ab <- read.csv(file.about, as.is=TRUE)

## Set the directory where XCMS will look for your data files

## The filepath for this directory should be given between the quotation marks

dir.raw <- "/project/hegemana/Mike Wilson/Species analysis" ab$Nfile <- file.path(dir.raw, ab$Nfile)

ab$Pfile <- file.path(dir.raw, ab$Pfile)

## Set the directory to store script output (your 'results' directory) and set up other filenames

dir.results <- "results"

file.peaksP <- file.path(dir.results, "peaksP.Rdata")

file.peaksN <- file.path(dir.results, "peaksN.Rdata")

file.groupsP <- file.path(dir.results, "groupsP.Rdata")

file.groupsN <- file.path(dir.results, "groupsN.Rdata")

file.intb <- file.path(dir.results, "intb.csv")

file.Rsq <- file.path(dir.results, "Rsq.csv")

file.scree <- file.path(dir.results, "scree.pdf")

file.screecsv <- file.path(dir.results, "scree.csv")

file.para <- file.path(dir.results, "para.pdf")

file.pc12 <- file.path(dir.results, "pc12.pdf")

file.pc12_modified <- file.path(dir.results, "pc12_modified.pdf")

## Make sure all the files in about.csv exist

## If a file does not 'exist' there is probably a discrepancy between the file name in about.csv vs. #the actual file name

file.exists(ab$Nfile) #Make sure all the (-) files exist

file.exists(ab$Pfile) #Make sure all the (+) files exist

############################################################

## Have XCMS get peaks from raw MS data and group them.

## Do not include files that are all noise (e.g. blanks), as the analysis will not work

## These steps can take time (depending on your computing power), so results are saved as R #data files. This section can be skipped later if you want to change the downstream analysis.

## Get positive peaks from raw MS data and group them

xset <- xcmsSet(ab$Pfile, method="centWave", ppm=10, peakwidth=c(5,50),

fitgauss=TRUE, verbose.columns=TRUE)

save(xset, file=file.peaksP)

xset <- group(xset, method="nearest", mzCheck=2, rtCheck=5)

save(xset, file=file.groupsP)

## Get negative peaks from raw MS data and group them

xset <- xcmsSet(ab$Nfile, method="centWave", ppm=10, peakwidth=c(5,50),

fitgauss=TRUE, verbose.columns=TRUE)

save(xset, file=file.peaksN)

xset <- group(xset, method="nearest", mzCheck=2, rtCheck=5)

save(xset, file=file.groupsN)

############################################################

## Merge (+) and (-) peak lists and create the final peak matrix

## Get positive peak list

load(file.groupsP)

Pintb <- groupval(xset, value="intb")

Pintb <- Pintb[,match(ab$Pfile, filepaths(xset))]

rownames(Pintb) <- paste("P", groupnames(xset), sep=".")

colnames(Pintb) <- ab$id

dim(Pintb)

## Get negative peak list

load(file.groupsN)

Nintb <- groupval(xset, value="intb")

Nintb <- Nintb[,match(ab$Nfile, filepaths(xset))]

rownames(Nintb) <- paste("N", groupnames(xset), sep=".")

colnames(Nintb) <- ab$id

dim(Nintb)

## Merge lists together

intb <- rbind(Pintb, Nintb)

dim(intb)

## Count the number of peaks in each raw data file and remove files with less than 100 peaks #from the analysis. Using files with < 100 peaks prevents completion of the analysis.

ab$npeaks <- colSums(!is.na(intb))

intb <- intb[,colnames(intb) %in% ab$id[ab$npeaks >= 100]]

dim(intb)

## Remove peaks that don't appear in every composite sample. This is a conservative data filter #that is designed to exclude noise peaks from the analysis.

comp <- intb[, ab$id[ab$group=="Composite"], drop=FALSE]

ncomp <- rowSums(!is.na(comp))

intb <- intb[ncomp >= ncol(comp), , drop=FALSE]

dim(intb)

## Remove composite samples. They are not needed for the rest of the analysis.

intb <- intb[, !colnames(intb) %in% ab$id[ab$group=="Composite"], drop=FALSE]

dim(intb)

## Remove composite peaks that are always or never present in the whole data set. We only want to look at things that are different between sample groups.

bsum <- rowSums(is.na(intb))

intb <- intb[bsum>0 & bsum<ncol(intb), , drop=FALSE]

dim(intb)

## Make all missing values in the peak matrix = 0 (e.g. if a peak was not present in a file, it will #get an area value of 0)

intb[is.na(intb)] <- 0

##Create 'intb.csv'. This is your peak matrix used for analysis

write.csv(intb, file=file.intb, na="")

############################################################

## Modify 'about.csv' so that it matches 'intb.csv'. This is important for tracking the meta-data in #‘about.csv’ correctly

intb <- as.matrix(read.csv(file.intb, row.names=1, check.names=FALSE))

intb.saved <- intb

## Remove composite files and files with < 100 peaks from "about.csv"

ab2 <- ab[match(colnames(intb), ab$id),]

ab2$group <- factor(ab2$group)

ab2.saved <- ab2

############################################################

## Optional - Using a subset of your peak matrix for analysis instead of the whole thing. This #allows you to change the files you want to compare (e.g. eliminating some groups from the #analysis) without having to create a new ‘about.csv. and run your files through XCMS a second #time. Only use one of the options below – isolating single groups or isolating multiple groups

## How to isolate a single group in your peak matrix, ‘intb’, for analysis

##The group (as given in ‘about.csv’) you want to analyze should be in the quotation marks

intb <- intb.saved[,grep("P. deltoides", ab2$group)]

## How to isolate multiple groups in your peak matrix, ‘intb’, for analysis

##The groups (as given in ‘about.csv’) you want to analyze should be in the quotation marks

##You can add another group into the comparison using '|'

use <- grepl("P. deltoides May", ab2$group) | grepl("P. deltoides June", ab2$group) use <- grep("P. deltoides", ab2$group)

## change both 'intb.csv' and 'about.csv' to include only those groups we want to analyze

intb <- intb.saved[,use]

ab2 <- ab2.saved[use,]

############################################################

##Analysis of your peak matrix, ‘intb.csv’. Only use one principle component analysis (PCA)

#option, presence/absence PCA or differential concentration PCA

## Prepare 'intb.csv' for presence/absence principle component analysis (PCA). This use of PCA #is designed to highlight differences between samples due characteristic and exclusive peaks.

Bx <- (intb>0)*1

Bx <- Bx[rowMeans(Bx) > 0 & rowMeans(Bx)<1,]

B <- scale(t(Bx))

## Prepare 'intb.csv' for differential concentration principal component analysis (PCA). This use #of PCA is designed to highlight differences between samples that are due to differing #concentrations of metabolites.

## Here we use a log_10_ transformation of area under peak. You can apply other transformations #as deemed necessary.

B <- t(log10(intb+1))

## Get principal components

Bs <- svd(B, nv=0)

Bpcs <- Bs$u %*% diag(Bs$d)

Bvar <- Bs$d^2/nrow(B)

colnames(Bpcs) <- paste("PC", 1:ncol(Bpcs), sep="")

## Create a scree plot showing the % variation explained by each principal component. Creates #both a .pdf and .csv file of scree plot results.

npc <- 30 # keep this many PCs for evaluation, could keep more or less

evar <- Bvar/sum(Bvar)

pdf(file.scree) #creates the scree plot as a pdf file in the 'results' directory

plot(evar[1:npc], ylim=c(0, max(evar)*1.05), yaxs="i")

dev.off()

write.csv(data.frame(pc=1:npc, var=evar[1:npc]), row.names=FALSE, file=file.screecsv)

## Create a .pdf plot of the first two principal components. You can change the principle #components plotted by modifying ‘PC2~PC1’ to your desired principle components

p <- xyplot(PC2~PC1, group=ab2$group, data=data.frame(Bpcs), auto.key=list(space="right"))

pdf(file.pc12)

plot(p)

dev.off()

## If there is an outlier in your PCA plot, determine which sample is it. Your definition of #‘outlier’ should be governed by your PCA plot, but for this example we have chosen a distance #magnitude of 30 to define ‘outlier’.

ab2[which(Bpcs[,"PC1"]>30),]

## Determine the row number of any outliers in ‘about.csv’

which(Bpcs[,"PC1"]>30)

## Create a new PCA plot that does not include the outlier(s). 19 is the outlier row number in #‘about.csv’ for this example.

p <- xyplot(PC2~PC1, group=ab2$group[-19], data=data.frame(Bpcs[-19,]), auto.key=list(space="right"))

pdf(file.pc12_modified)

plot(p)

dev.off()

## Create a parallel plot of first 5 principle components

p <- parallelplot(~Bpcs[,1:5], group=ab2$group[-19], horizontal=FALSE, common.scale=TRUE,

auto.key=list(space="right")) #can exclude outliers by modifying ab2$group to ab2$group[-"outlier row"]

pdf(file.para)

plot(p)

dev.off()

## Determine if any peaks are strongly correlated with the principle components, and get the R^2^ #values for this correlation. This will essentially tell you which peaks are causing any group #segregation seen in the PCA plot. The default is to use only the first 3 principle components.

npc2 <- 3

pcs <- Bpcs[,1:npc2]

sumpcs <- colSums(pcs^2)

Rsq <- colSums(cor(pcs, B)^2*sumpcs)/sum(sumpcs)

## Determine the presence/absence of a given peak by sample. This is designed to give you an #overview of peaks that are characteristic of/reproducible in a given sample group.

## Outliers can be excluded by modifying ab2$group to ab2$group[-"outlier row"]

ii <- split(1:ncol(Bx), ab2$group)

count <- sapply(ii, function(i) rowSums(Bx[,i,drop=FALSE]))

## Output peak correlation and sample appearance information (ordered by highest R^2^ value) #into a .csv file. This gives you a file that can be manipulated in Microsoft Excel to find #reproducible and characteristic peaks that are contributing to group segregation in the PCA plot.

out <- cbind(count, Rsq)

out <- out[order(out[,"Rsq"],decreasing=TRUE),]

write.csv(out, file=file.Rsq)
